# Supplementary material for: Are carbon emissions trading and green financial instruments synergistic? -Comprehensive quantitative research based on content analysis
Source: PLoS One. 2024 Mar 7;19(3):e0298601. doi: 10.1371/journal.pone.0298601 (PMC10919601; doi:10.1371/journal.pone.0298601)
Supplement: S1 Data — (ZIP) [file pone.0298601.s001.zip › Data/地级市Z_F工作报告.zip!/╕ú╜¿/╧├├┼╩╨/└┤╫╘╣1⁄2╓┌║┼í╛┬φ┐╦╩2╛▌═°í┐_2013.docx]

各位代表:

　　现在，我代表市人民政府向大会作政府工作报告，请予审议，并请市政协各位委员和其他列席人员提出意见。

　　一、2012年工作回顾

　　2012年，我们在市委的坚强领导下，以邓小平理论、“三个代表”重要思想、科学发展观为指导，以学习贯彻党的十八大精神为动力，坚持稳中求进、好中求快，实现经济社会健康协调发展。预计全年地区生产总值2822亿元，增长12%；规模以上工业增加值1060亿元，增长12.1%；全社会固定资产投资1360亿元，增长20%；财政总收入739亿元，增长13.4%，其中地方级财政收入423亿元，增长14.1%；社会消费品零售总额880亿元，增长10.3%；城镇登记失业率3.5%；居民消费价格指数102.2；城乡居民收入与国内生产总值同步增长；节能减排目标预计可以完成。

　　一年来的主要工作和成效是:

　　（一）着力实施综改方案，重大平台建设成效显著

　　面对中央赋予厦门新的历史使命，我们加强组织领导，坚持统筹规划，建立协调机制，狠抓责任落实，全力推进重点领域改革、重要政策争取、重大平台建设。

　　综合配套改革有序推进。制定并实施综改三年行动计划，56个部门分别出台工作措施。市对区财政体制改革、医药卫生体制改革、事业单位分类改革、国有企业改革稳步推进。大嶝对台小额商品交易市场免税额度由3000元提高到6000元，“营改增”试点、发布海峡航运指数等20项政策获批实施。省政府出台实施意见，支持厦门建设东南国际航运中心和两岸区域性金融服务中心。两岸新兴产业和现代服务业合作示范区发展规划上报国务院。规划建设大陆对台贸易中心等重大平台。

　　东南国际航运中心加快建设。推动客运与货运并举、软件与硬件并重、港口与产业同兴、腹地与中转结合，大力发展航运物流综合服务体系。厦门航运交易所、东南国际航运仲裁院、福建电子口岸入驻。远海自动化码头、中心总部大厦等重大项目进展顺利。海沧保税港区二期封关运作。东渡邮轮母港规划启动，亚洲最大邮轮“海洋航行者号”成功靠港。内支线网络进一步完善，陆地港建设取得进展。港口集装箱吞吐量突破700万标箱，增幅居全国前列。厦门成为全国唯一海港空港“国际双创卫”城市。

　　两岸区域性金融服务中心建设进展顺利。围绕“高起点、快启动、早见效”，创新体制机制，加快项目集聚。厦门农商银行等20家金融机构开业。赛富创投基金等112个项目落户。海西金谷广场开工，将建成区域性金融要素交易平台。浦发银行在厦设立离岸业务中心，厦门银行等6家金融机构在异地设立分支。我市与台湾多家银行签订人民币清算代理协议，人民币结算额增量居全国首位。

　　（二）着力稳增长调结构，经济发展方式加快转变

　　进一步加大政策扶持力度，积极推动经济发展更多依靠科技进步、劳动者素质提高、管理创新驱动。

　　自主创新能力进一步提高。火炬高新区国家级孵化器面积超过50万平方米，在孵企业1000多家。新增国家“千人计划”专家15人。新设中科院海西研究院稀土研究所等研发机构27家。厦门南方海洋研究中心挂牌。生物与新医药特色产业基地通过国家认定。世界第一支重组戊肝疫苗获批生产。河豚毒素完成二期临床试验。新认定4家省级企业工程技术研究中心。新增2家国家创新型试点企业。参与起草国家、行业技术标准58项。国家标委会组织达11家。专利申请9246件，授权7178件。

　　工业转型升级步伐加快。大力鼓励企业提升设备现代化、生产自动化水平，稳定产品质量，提高市场竞争力。保沣实业、正新集美厂等企业自动化生产线投产。天马微电子、开发晶照明等企业投入运营。厦船三期等项目开工，山东重工重组林德叉车和丰泰汽车。新增联想移动、冠捷、烟草工业3家产值超百亿元企业。新认定高新技术企业169家，达到772家，实现产值2300亿元，增长20%。厦门钨业列入全省首个国家循环经济标准化试点单位。成功承办工业设计领域首个国家级奖项展评。德国红点设计机构首次在我市举办国际设计营商周。全年完成工业投资 260亿元，增长12.6%。单位电力产值为全省平均值的近2倍。

　　现代服务业水平不断提升。服务业增幅居全省首位，对经济增长贡献率超过60%。海峡电子商务创业园建成使用，电子商务企业快速成长。石油交易等专业市场形成规模，四大营运中心新引进总部155家。离岸服务外包合同金额和执行金额分别增长67%和37%。美亚柏科超级计算中心正式运营。软件园三期加快推进。电信、移动、联通动漫基地齐聚厦门，软件和信息服务业销售收入461亿元。全年共举办展览活动160场，展览面积138万平方米，外来商业会议2300场，参会人数50万人次。会展中心三期建成，总展览面积10万平方米。威斯汀、凯宾斯基等高端酒店开业。旅游人气和游客满意度指数居全国前列。接待境内外游客4016万人次，增长14%，旅游总收入531亿元，增长17%。中山路名列第四届“中国历史文化名街”榜首。

　　帮扶企业有力有效。落实结构性减税缓税、涉企收费减免等政策措施，全年为企业减负23亿元。出台促进工业生产、稳定出口增长的相关措施，加大对中小微企业扶持力度，鼓励企业增产多销，全年提供财政扶持资金超过36亿元。实行重点企业“一企一策”，帮助摆脱困境，转型升级。七成规模以上工业企业实现盈利，工业经济效益指数比年初回升16个百分点。出台加快创新体系建设、加快海洋经济和旅游产业发展的实施意见，营造更佳的发展环境。

　　（三）着力统筹城乡发展，一体化同城化进程加快

　　大力推进跨岛发展战略，合理布局城市人口和生产要素，不断提升城市综合承载能力和对外辐射能力。

　　新城建设全面提速。完善四大新城组织管理、开发建设、投融资等工作机制，相继启动一批工程，完成投资430亿元。集美、海沧、同安、翔安新城分别完成建筑面积198、174、113、70万平方米。厦大翔安校区正式开学，当年入驻5000多名学生。洋唐保障性安居工程、洪坑安置房初具规模。同集路改造步伐加快，海翔大道二期、海沧隧道连接线、滨海东大道开工建设，集美北大道等干线交通路网建成通车。

　　岛内提升步伐加快。严格控制城市建设规模，降低开发强度，拆除违章建筑70万平方米。优化提升沿街建筑立面和夜景工程。继续治理筼筜湖。启动火车站改造。坚持“退二优三”，湖里老工业区逐步转型。完成849辆出租车“油改气”，对近600条道路定期清洗，有效减少粉尘。提升绿化彩化水平，新建一批林荫道和街心公园。

　　厦漳泉同城化扎实推进。编制完成总体规划和9个专项规划，持续推进57个同城化项目。龙厦铁路、厦安高速建成通车，厦漳跨海大桥全线贯通。长泰枋洋水利枢纽工程通过国家环评审查。九龙江北溪引水左干渠改造工程投用。完成厦漳港口一体化整合。海投长泰科技园、火炬永春产业园建设启动。

　　“三农”工作成绩突出。全面落实强农惠农富农政策，拓宽农民增收渠道，实现转移就业2万人，农民人均纯收入居全省首位。同安国家农业科技园加快建设。农副产品与食品加工业实现产值288亿元，增长16%。建设1.5万亩蔬菜种植直控基地。莲花水库等水利工程顺利推进。除险加固2座中型水库。完成汀溪水库上游5个行政村连片整治。完成凤南和莲花片区自来水管网改造，解决6万人用水困难。汀溪、新圩、灌口、东孚小城镇建设进展顺利。

　　（四）着力加强外经外贸，开放型经济水平稳步提升

　　充分利用国内外两种资源、两个市场，不断提升招商引资水平，加快转变外贸增长方式。

　　“三维”招商取得成效。突出抓龙头、铸链条，新增世界500强项目11个，新设立外资项目330个，实际利用外资25.3亿美元。央企对接合作项目8项，16个在建项目进展顺利。对接民企项目194项，总投资808亿元。成功举办第16届“投洽会”，参展规模进一步扩大，厦门团外资签约项目40.5亿美元。

　　对外经贸平稳发展。面对企业订单不足、成本升高等困难，深入实施以质取胜和市场多元化战略。全面实行海关进出口分类通关改革，在全国率先实现进出口货物检验检疫电子监管。全年进出口总额744亿美元，增长6%，其中出口452亿美元，增长6%。新建成外贸公共服务平台8个。外贸综合竞争力位居全国百强城市第5位。

　　对内对外交流合作取得新进展。接待外国团组258批次。与加拿大列治文市缔结友好城市。开通厦门至柬埔寨直航航线。厦航加入天合联盟，进一步提升了国际影响力。成功举办国际海洋周、南洋文化节。“厦门号”帆船完成环球航行。爱乐乐团出访俄罗斯。海外华侨华人社团联谊工作得到拓展。闽西南五市等区域协作得到加强，援疆援藏援宁等对口帮扶取得新成效。

　　（五）着力发挥对台优势，两岸交流合作更加紧密

　　作为全国唯一深化两岸交流合作综改试点城市，我们致力先行先试，不断开创对台工作新局面。

　　涉台服务更加优化。维护台胞合法权益，帮助台企解决困难。全市金融业为台企提供1400亿元人民币授信额度。聘请台胞担任陪审员和检察联络员，设立涉台海事审判庭、基层法院涉台法庭、台商协会法律援助站，率先备案管理台湾经贸社团在厦设立机构。8所一级达标高中设立台生班。

　　经贸往来更加密切。全市新批台资项目149个，合同利用台资4.2亿美元(含第三地)；对台贸易77.8亿美元，台湾水果进口量保持大陆各口岸第一。欣贺服饰等6家企业入台投资。海峡两岸图书物流中心投用。厦金航线开通夜航，赴金门旅游实现当天办证、当天成行。大陆居民经厦赴台旅游16万人次。开通厦门至澎湖包机航线和两岸海上快件运营。厦金海底光缆竣工，两岸经由第三方通信的历史将宣告结束。

　　交流合作更加深入。成功举办第四届海峡论坛，台湾中南部、中下层、中小企业等参会代表超过万人。台交会、文博会、郑成功文化节、保生慈济文化旅游节等重大活动水平和层次提升。与台湾岛内重要人士的交流交往取得突破。新设台中市海交会办事处等12家两岸办事机构。成功举办两岸海上联合搜救演练。厦台科技、教育等领域交流合作取得新进展。

　　（六）着力坚持以人为本，和谐社会建设深入推进

　　优先保障民生投入，优先办好民生实事，不断化解民生难题，促进社会更加和谐，人民生活持续改善。

　　社会事业加快发展。实施50件为民办实事项目。新建、改扩建公办幼儿园23所，新增学位6600个。完成13所中小学建设，新增学位1.2万个。义务教育学校标准化建设达100%。面向全国招收1264名中小学幼儿园教师。在全省率先实行中等职业教育免学费、农村义务教育阶段学生营养膳食补助、统一全市中小学的生均公用经费。厦门理工学院通过本科教学合格评估。中山医院内科综合病房楼竣工，口腔医院新院投用，全市新增病床位近千张。提前三年将基本公共卫生服务经费提高到年人均40元。持续稳定低生育水平，出生人口政策符合率95.7%。鼓浪屿申遗等进入我国预备名单。《蝴蝶之恋》荣获全国“五个一工程”奖。全市镇（街）、社区配齐综合文化站、文化活动室、电子阅览室。闽南大戏院等项目竣工。我市运动员在伦敦奥运会夺得1金2铜。厦门国际马拉松实现国际田联路跑金牌赛事“五连冠”。荣获全国双拥模范城“八连冠”。防震、气象、方志、档案、人防海防、民族宗教、妇女儿童、老龄、红十字、慈善等工作取得新进步。

　　民生保障更加有力。新增就业20.3万人，荣获全国创业先进城市。企业退休人员基本养老金上调255元，增幅居全国第二。城乡居民医保每人每年财政补助标准提高到360元，提前三年达到国家标准。城镇职工和城乡居民大病保险的报销比例提高到95%和75%。对困难残疾人发放每人每月300元生活补贴。为智障儿童设立集教育、康复、科研于一体的心欣幼儿园竣工。在三级公立医院推行先诊疗、后结算。城乡居民基本医疗保险门诊和住院报销比例提高到50%和75%。城市、农村低保标准每人每月上调65元和70元。建立覆盖全市的农产品质量预测预警监测点。继续定点供应平价农副产品，建成平价商店57家。开工建设2.9万套保障房，完成5073户配租配售。新建、启用一批停车楼和路边停车位，增加停车位近2万个。新开通与优化公交线路68条，新增与更新公交车450辆。

　　社会管理不断创新。全面启动综合警务改革，路面见警率、管事率明显提高。持续开展“清网行动”和“两抢一盗”等专项整治。推进社区网格化、信息化、精细化、人性化服务管理，海沧区成为全国社区管理和服务创新实验区。顺利完成村（居）组织换届选举。全面开展市区镇（街）领导干部接访、下访活动，化解矛盾纠纷。开展“安全生产年”活动，落实安全生产责任制。出入境管理处被国务院授予“模范出入境管理处”荣誉称号。

　　生态环境持续改善。全面开展生态市建设，落实城乡环境综合整治“点、线、面”攻坚计划，造林绿化2.8万亩，改造提升4785亩。开展水土流失治理。会展中心岸线人造沙滩工程、集杏海堤开口改造顺利完工。垃圾密闭化清运及时率、无害化处理率达99.5%。东部垃圾焚烧发电厂投入试运行。顺利通过再创国家环境保护模范城市复核，主动发布PM2.5检测值，空气质量优良率100%。获评国家可再生能源建筑应用示范城市。

　　（七）着力政府自身建设，行政效能水平不断提高

　　自觉接受人大及其常委会监督，依法执行人大决定决议。支持人民政协履行政治协商、民主监督、参政议政职能。全年办理人大议案9件、建议314件，满意率98.5%；办理政协提案562件，满意率97.2%。扎实推进依法行政，连续6年获得全省依法行政绩效考评第一名。提请市人大常委会审议法规草案4件，制定、修改规章5件。市政府部门“三定”工作全面完成。深入开展重点领域、重点项目、重点资金审计和监察，廉政建设和反腐工作得到加强。开展“下基层、解民忧、办实事、促发展”和“深化效能建设年”活动，治理庸懒散，建立直接联系群众服务基层常态机制。75个行政事业单位进驻市政务服务中心，为市民提供600多项“一站式”服务。电子政务云投入运行。厦门蝉联全国服务型政府第一名，再次名列全国城市文明指数测评首位。

　　各位代表！这些成绩的取得，是在党中央、国务院和省委、省政府与市委的坚强领导下，全市人民齐心协力、艰苦奋斗的结果。在此，我谨代表市人民政府，向全市人民，向给予政府工作大力支持与有效监督的人大代表和政协委员、各民主党派、工商联、各人民团体、无党派人士、离退休的老同志和社会各界人士，向中央和省驻厦单位、驻厦部队、武警官兵和公安干警，向来厦的投资者和劳动者，向关心支持厦门发展的港澳同胞、台湾同胞、海外侨胞和国际友人，表示衷心的感谢和崇高的敬意！

　　同时，我们也清醒地看到，当前发展还面临不少困难和问题，主要是:资源与环境约束日益趋紧，培育新的经济增长点、推动产业结构优化升级任务繁重；经济总量偏小制约了中心城市集聚辐射功能的有效发挥；教育、文化、医疗等公共服务需求快速增长，与供给不足之间的矛盾较为突出；停车难、交通拥堵现象有待缓解；一些领导干部和工作人员作风不实，工作水平和能力有待提高。我们要正视这些问题，努力加以解决。

　　二、2013年工作安排

　　2013年是贯彻落实党的十八大精神的开局之年，是为全面建成小康社会奠定坚实基础的重要一年。做好今年工作，既要认真研究分析当前国内外形势，充分把握可以大有作为的重要战略机遇期，又要进一步增强忧患意识，看到各种不利因素，不回避矛盾，不掩盖问题，从而坚定必胜信心，积极作为，始终保持经济社会持续健康发展。

　　政府工作的总体要求是:深入学习和贯彻落实党的十八大精神，坚持以邓小平理论、“三个代表”重要思想、科学发展观为指导，按照党中央国务院、省委省政府和市委工作部署，紧紧围绕主题主线，以提高经济增长质量和效益为中心，坚持稳中求进，全面实施跨岛发展战略，进一步深化改革、扩大开放，拓展对台交流合作，加快产业转型，壮大实体经济，建设创新城市，拓展城市空间，构建和谐社会，推进生态文明建设，实现科学发展新跨越。

　　经济社会发展主要预期目标为:地区生产总值增长11%，力争12%；规模以上工业增加值增长 12%；全社会固定资产投资增长20%；外贸出口增长7%；实际利用外资与上年持平；地方级财政收入增长13%；城乡居民收入增长12%左右；城镇登记失业率控制在4%以内；居民消费价格指数103.5左右；人口自然增长率控制在9‰以内；完成国家和省下达的节能减排指标任务。

　　实现上述目标，必须扎实做好以下工作:

　　（一）围绕城市发展定位，构建现代产业发展新体系

　　科技创新是发展的根本动力，产业转型是发展的现实路径。要大力发展高端产业和产业高端，筑牢实体经济基础。

　　大力发展先进制造业。以提升企业自动化水平和品牌创建能力为重点，用新技术、新工艺、新设备抬高制造业起点。坚持项目集结园区、产业集群发展、资源集约利用。促进天马微电子等项目尽快达产，加快ABB整体搬迁、中船重工七二五所等项目建设。利用高新技术和先进适用技术改造提升传统优势产业。完善市、区两级挂钩联系企业工作机制。积极扶持成长型中小企业和科技型初创企业。实施品牌战略和标准化战略，争创首批全国质量强市示范城市。

　　加快发展现代服务业。要围绕拓展新领域、发展新业态、培育新亮点，突出发展航运物流、金融商贸、会展旅游、科研文教等产业，大力推进供应链管理、电子商务、服务外包、信息、会计、咨询、法律服务等新兴服务业。推进前场铁路大型货场和航空港物流园区建设。大力发展高端商贸业。培育、引进更多品牌展会和赛事活动。打造电子商务集中区，把电子商务作为扩大内需的新战场，促进商业模式由传统向现代转型。完善扶持政策，积极发展总部经济。

　　强化创新驱动。以建设国家创新型试点城市为引领，构建以企业为主体、市场为导向、产学研结合的区域创新体系。鼓励企业开展新技术新产品新工艺的研究应用。以关键核心技术的突破，带动新一代信息技术、生物与新医药、新材料等战略性新兴产业。加强各类研究机构、公共创新平台建设。加快筹建微电子产业园、台湾创新科技园。完善科技服务体系，推动科技与金融结合。加强国家知识产权示范城市建设。推进“海纳百川”人才计划，以重大项目、创新平台为载体，大力引进创新领军人才和创业团队。

　　加快发展海洋经济。厦门因海立市、因海开放、因海发展。要加快海洋开发，建设海洋经济强市。做精海洋研究，扶持厦门南方海洋研究中心发展，使之成为我国海洋基础研究、高新技术与成果转化的重要基地。办好国际海洋周，争取创建国际海洋组织。做大海洋贸易，巩固传统市场，开辟新兴市场，拓展内陆腹地，发展内支线，扩大内贸中转，保持在全省外贸的龙头地位。做优海洋运输，推进港航基础设施建设，建成远海全自动化码头。做强海洋产业，大力发展海洋生物医药、邮轮游艇等产业集群。

　　加快发展旅游产业。旅游业是厦门经济发展的优势产业，是扩大内需、拉动消费的引擎。做足滨海休闲旅游，大力发展游艇、帆船、邮轮等海上旅游，推进环东海域高端酒店群等项目的招商与建设。做响海峡旅游品牌，探索与金门共建“厦金旅游协作特区”。扩大赴台、赴金马澎个人游。加快鼓浪屿申遗，强化文化遗产核心要素保护。促进华强科技文化产业基地开业。

　　（二）围绕增创特区新优势，全面深化改革扩大开放

　　以更大的决心和勇气深化改革，用创新的思维和举措开辟发展路径，为发展提供强劲的动力活力源泉。

　　加快重大平台建设。东南国际航运中心要尽快完成发展规划，突出核心区建设。推进东渡港区传统业务搬迁，加快建设邮轮母港。集聚航运要素，研究出台揽货、扩展航线、船舶注册等相关政策。发挥厦门港整合后的优势，实行同港同策。加快高崎机场三期工程建设和翔安新机场前期工作，支持开辟新的洲际航线，积极发展航空制造等产业。两岸区域性金融服务中心要以集聚金融资源、推进金融创新为重点，加快金融要素市场建设，拓展集聚辐射功能。推动更多企业通过上市、发债、并购重组等方式，拓宽融资渠道。鼓励符合条件的企业设立小额贷款公司。积极发展跨境人民币业务和离岸金融业务。扎实推进保险改革和发展。两岸新兴产业和现代服务业合作示范区要全力争取产业准入和优惠目录获批，抓紧核心区各个专项规划，鼓励龙头项目落户，推动节能环保、文化创意、研发设计等产业发展。

　　加快外贸转型升级。适应经济全球化新形势，加快转变对外经济发展方式，推动开放朝着优化结构、拓展深度、提高效益方向转变。坚持出口与进口并重，打造以技术、品牌、质量、服务为核心的出口竞争新优势，促进加工贸易向产业链高端延伸，做大做强优势产业出口基地。整合优化海关特殊监管区域，力争落实启运港退税。大力发展流通型外贸出口，积极吸引异地货源。进一步完善电子口岸建设，加强区域物流发展合作。扩大原材料等大宗商品进口。积极鼓励企业走出去，增强国际化经营能力。

　　提升“三维”招商水平。围绕提高利用外资综合优势和总体效益，瞄准世界500强、跨国公司和大型央企、民企，以重要平台和产业功能区为载体，细化重点产业集群发展规划，强化产业链招商，挖掘一批产业链关联项目，策划生成一批带动力强的好项目。继续做好对口支援西藏、新疆、宁夏等工作。

　　（三）围绕增强中心城市功能，加速一体化同城化

　　把现代化国际性理念融入到城市建设全过程，始终按照“四高”要求，提升城市品位，增强集聚辐射功能。

　　加快新城建设。坚持四大新城各自特色定位，突出生态宜居，丰富历史、民俗、文化内涵，体现差异化发展。尤其是集美新城，要彰显嘉庚建筑风格。坚持新城建设与产业发展、社会事业并重，抓紧配套完善水、电、路等重大基础设施，建成一批标志性工程；推进一批拉动力强的生产性项目；建设一批学校、医院等重大民生项目；完善一批酒店、娱乐、商业网点等公共配套设施。以新城吸引产业发展，以产业支撑新城建设，使每一项规划建设都经得起历史的检验。

　　持续提升岛内。控制开发强度，降低建设容量，优化土地使用，完善功能配套，提升环境品质。继续推动五缘湾、湖边水库等片区开发建设，高标准打造东部新区。加快湖里老工业区改造步伐，推进厦港、枋湖等片区改造，支持老旧住宅区整治改善。开工建设轨道交通1号线和海沧隧道，做好轨道交通沿线片区的征地拆迁和综合开发，合理利用地下空间，构建地铁新兴商圈。

　　加快厦漳泉同城化。对接交通路网，协调布局枢纽场站。加快建设厦漳泉城市联盟线等通道，建成厦深铁路（厦门段），加快城际轨道交通前期工作。协调推进信息通信一体化，争取固定电话升位并网、统一区号和资费。推进九龙江流域综合整治，推进长泰枋洋水利枢纽库区移民安置工作。研究产业融合发展新机制，力促特区政策向大都市区延伸拓展。

　　做好城市化过程中的“三农”工作。城乡发展一体化是解决“三农”问题的根本途径。要让广大农民在城市化进程中分享成果。加快建设特色农业高新技术产业化示范基地；扶持农业产业化龙头企业，提升都市现代农业发展水平。加大农村公共服务和基础设施投入，加快气象雷达升级工程建设，推进城乡公交、路灯、绿化、环卫等一体化。推进旧村改造、老区山区村建设和移民造福工程。深化“村改居”集体资产改制，用好农村集体预留发展用地等扶持政策，大力发展农村集体股份合作经济，加强村集体项目招商招租和运营管理，促进农民持续增收。

　　（四）围绕前沿平台优势，深化对台交流合作

　　进一步发挥五缘优势，用好、用活、用足国家赋予的对台先行先试政策，提高对台交流合作水平。

　　深化经贸合作。积极参与两岸产业“搭桥计划”，建立产业深度对接机制。加快厦门台商总部大厦建设，吸引更多台企来厦设立区域总部、运营中心、研发中心、采购中心、物流中心。推进闽台文化产业园建设。做大做强大陆对台贸易中心，加快建设两岸航空冷链业务中转试点、输台药材集散中心。

　　便利直接往来。争取扩大一年有效多次往返签注通行证适用范围和对象，进一步争取更多赴台交流审批权限，促进往返两岸手续更加便捷。建成五通码头改扩建工程。力争海峡邮轮航线常态化。吸引更多民众经厦金航线往来两岸。

　　加强交流交往。精心办好第五届海峡论坛、文博会等涉台重大活动。继续深化与台中基层党政交流，推动两岸红十字会等社团组织对口交流合作。继续争取国家部委来厦设立基地，推动两岸相关团体、台湾地区县市来厦设立办事机构，筹备设立海峡两岸社区交流等专业协会。

　　（五）围绕实现永续发展，加快生态文明建设

　　把生态文明建设贯穿于经济社会发展各方面和全过程，推进绿色发展、循环发展、低碳发展，建设美丽厦门。

　　推进节能减排。加快国家低碳城市试点建设，落实节能目标责任制，抓好节能重点工程和示范工程，按绿色建筑标准设计建设保障性住房。创建全国公共建筑节能改造示范城市，严格实施固定资产投资项目节能评估和审查制度。完善主要污染物减排激励和约束机制，加速淘汰落后产能和工艺。推行企业清洁生产，倡导清洁施工，大力发展循环经济。提高供地门槛，处置闲置土地，推动集约发展。

　　加强环境保护。积极创建国家生态市，加强饮用水源保护、环境安全、流域污染治理等重点工作。加快岛外10条溪流治理，加大环岛路沿线和鼓浪屿截污工程改造力度。加快高集海堤开口改造主体工程建设，做好海域清淤、岸线整治和海漂垃圾清理。加快国家级海洋公园建设，开展滨海湿地和沙滩修复，强化白海豚保护，建设全国海洋生态文明示范区。加快农村污水治理，改善地表水环境质量。推动西部垃圾焚烧发电厂投入运营。强化道路清洗工作，遏制土方车跑冒滴漏，持续提高空气质量。

　　提升绿化美化。创建国家生态园林城市、国家森林城市。提升城市园林绿地质量，继续创建绿色乡镇和绿色村庄。推动高速、高铁等道路沿线绿化配套及景观提升。治理水土流失，防治地质灾害，开展林分补植和结构提升，建设绿色屏障。加快建设集美湖、海沧大屏山、同安中洲滨海、翔安后山岩等公园绿地。

　　（六）围绕提高公共服务水平，加快发展社会事业

　　按照城乡一体化、均衡化的要求，完善公共服务体系建设，提高城市文明水平。

　　办好人民满意的教育。大力发展学前教育，均衡发展义务教育。新建18所公办幼儿园、34所中小学。扶持规范民办幼儿园。加大对岛外学校的扶持。提高进城务工人员子女接受公办义务教育的比例。持续发展职业教育，加强与产业发展相适应的专业学科体系建设。做好厦大、集大、华大等省部属高校共建工作。支持厦门理工学院进入省重点建设高校行列。加快城市职业学院集美校区建设。加强师德建设，提高教师队伍素质。办好集美学校百年校庆，弘扬嘉庚精神。

　　不断提高市民健康水平。在公立医院实施药品“零差价”，降低大型医疗设备检查治疗费用，减轻群众看病负担。实施慢病管理一体化，推进建立分级诊疗制度。将全部行政村卫生室纳入国家基本药物制度实施范围。支持市级医学中心和重点专科向专科医院发展。推进翔安医院、五缘医院、海沧医院建设，加快形成多元化办医格局。继续稳定低生育水平，提升出生人口素质，丰富计划生育优质服务内涵。

　　增强文化整体实力和竞争力。保护和发掘城市历史文脉，彰显闽南文化特质。创建国家公共文化服务体系示范区、全国版权示范城市。加快重大公共文化工程和文化项目建设，筹建厦门大剧院。强化特色文化品牌建设，继续创作生产一批精品剧目，积极引进高质量院线和演出团队，将闽南大戏院打造成精品剧院。广泛开展群众性文体活动，办好中国国际钢琴比赛、全国第四届中小学生艺术展演和国际马拉松赛等活动。支持创意设计、影视动漫、文化旅游、数字内容等文化创意产业发展，培育一批有竞争力的文化龙头企业。

　　深化新一轮文明城市创建。加强社会主义核心价值体系建设，广泛开展“讲文明树新风”活动，提高公民道德素质和社会文明程度。推进道德讲堂建设，加强道德模范宣传，深入开展道德领域突出问题专项教育和治理活动。推进未成年人思想道德建设，抓好未成年人心理健康辅导站、乡村少年宫等项目实施。健全志愿服务体系，推动志愿服务工作。

　　（七）围绕保障和改善民生，加强和创新社会管理

　　坚持以人为本、民生优先，顺应人民群众过上更好生活的新期待，努力建设民生幸福城市。

　　推动实现更高质量的就业。鼓励多渠道多形式就业，促进创业带动就业，为每一个有劳动能力的人创造就业机会。完善技工教育和职业培训体系，提高城乡劳动者创业能力。做好以高校毕业生为重点的青年就业工作，促进农村转移劳动力、城镇困难人员就业。适时调整最低工资标准，完善企业工资正常增长机制。严格劳动合同制度，妥善处理劳资纠纷，构建和谐劳动关系。

　　提升民生保障水平。提高城乡居民养老保险基础养老金、企业退休职工养老金、城乡低保标准；提高城乡居民医疗保险的筹资标准和财政补助标准。加快社会养老服务体系建设，支持社会力量兴办公益性养老服务机构。完善困难群体动态生活补贴机制。提升食品安全和农残检测技术，加强食品流通领域管理。完善粮食安全保障体系，加快蔬菜生产基地建设。加强保障性安居工程建设与管理。优先发展城市公共交通，提升交通智能化水平，增开公交线路，扩大岛外公交覆盖面。推进海沧、翔安客运枢纽工程建设。妥善处理好重大项目实施与出行畅通的关系。整治违章建筑，推进城市精细化管理。

　　维护安定稳定。深入推进平安厦门建设，健全突发公共事件预警和应急处置机制、社会稳定风险评估机制，筑牢社会治安防控体系，依法严厉打击各种刑事犯罪，争创全国综治优秀地市“三连冠”。落实安全生产责任制，深入开展道路交通安全综合整治。排查与整治消防、电梯安全等重点领域隐患。推进安全社区创建，提升社会安全管理水平。畅通和规范群众诉求表达渠道，维护群众合法权益。重视舆情疏导，发挥公共媒体通达社情民意和化解社会矛盾的作用。

　　三、持之以恒建设服务型政府

　　建设职能科学、结构优化、廉洁高效、人民满意的服务型政府，是时代的召唤，人民的期待。

　　创新政府管理。必须解放思想、深化改革，把握时代发展要求，不懈探索经济特区建设发展规律。必须简政放权，推动政府职能向创造良好发展环境、提供优质公共服务、维护社会公平正义转变。必须深化行政审批制度改革，加快推行审批服务全程代理制，扩大网上审批范围，进一步减少审批事项和环节，提高办事效率。必须创新行政管理方式，完善激励考评机制，加强政策执行和任务落实情况督查，提高政府公信力和执行力。

　　切实改进作风。良好的政风是推动工作、实现目标的重要保证。要认真落实改进工作作风“八项规定”，坚持调查研究，深入基层，深入群众，及时为群众排忧解难。下决心改进文风会风，少开会、开短会，开解决实际问题的会；少讲话、讲短话，讲群众听得懂、记得住、用得上的话；少发文、发短文、发简明扼要管用的文。着力整治庸懒散奢等不良风气，讲求实际，注重实效，不搞短期行为，不做表面文章，致力于打基础、利长远、惠民生，多办人民满意的好事实事。坚持主动作为，不等不靠，以优良的政风奋力开拓经济特区更为广阔的发展前景。

　　推进依法行政。认真执行市人大及其常委会的决议和决定，主动接受人大法律监督、工作监督和政协民主监督。切实做好人大代表建议、意见和政协委员提案办理工作，定期通报情况。认真听取各民主党派、工商联和无党派人士意见，充分发挥工、青、妇等人民团体的作用。坚持问政于民、问需于民、问计于民，加大政务公开力度。坚持依法行政，加强政府立法工作和行政执法监督，提高依法科学民主决策水平，增强运用法治方式深化改革、推动发展、化解矛盾、维护稳定的能力，全面建设法治政府。

　　强化廉政建设。清廉是人民对政府的基本要求，是政府正确履行职责的重要保证。要坚持克己奉公、勤勉尽责，做人民满意公仆。坚持用制度管权管事管人，从重点领域、重点环节入手，狠抓惩治和预防腐败体系建设，确保廉政要求贯穿政府工作全过程，做到干部清正、政府清廉、政治清明。深入开展执法监察、效能监察、廉政监察，加强对政府重点投资项目、重点资金支出的监管和跟踪审计，严厉查处违纪违法案件。坚决反对铺张浪费，把财政资金用在刀刃上，严格控制“三公”消费，推进公务消费制度改革和信息公开，努力建设阳光政府、廉洁政府。

　　各位代表！展望新的一年，我们激情满怀、信心百倍。我们要紧密团结在以习近平同志为总书记的党中央周围，以更加昂扬的斗志、更加饱满的热情、更加扎实的作风，解放思想，凝心聚力，努力推进厦门经济特区科学发展新跨越，为早日建成现代化国际性港口风景旅游城市而努力奋斗！
